# Patients *embodied* and *as*-*a*-*body* within bedside teaching encounters: a video ethnographic study

**DOI:** 10.1007/s10459-016-9688-3

**Published:** 2016-05-31

**Authors:** Christopher Elsey, Alexander Challinor, Lynn V. Monrouxe

**Affiliations:** 1 Primary Care and Population Sciences (PCPS), University of Southampton, Southampton, UK; 2 Institute of Medical Education, School of Medicine, Cardiff University, Cardiff, Wales, UK; 3 Chang Gung Medical Education Research Centre (CG-MERC), Chang Gung Memorial Hospital, 5. Fu-Hsing St., Kuei Shan District, Taoyuan City, 333 Taiwan, ROC

**Keywords:** Doctor, Medical student, Nonverbal communication, Patients, Qualitative research, Teaching methods, United Kingdom

## Abstract

Bedside teaching encounters (BTEs) involve doctor–patient–student interactions, providing opportunities for students to learn with, from and about patients. How the differing concerns of patient care and student education are balanced in situ remains largely unknown and undefined. This video ethnographic study explores *patient involvement* during a largely *student*-*centric* activity: ‘feedback sequences’ where students learn clinical and practical skills. Drawing on a data subset from a multi-site study, we used Conversation Analysis to investigate verbal and non-verbal interactional practices to examine patients’ inclusion and exclusion from teaching activities across 25 BTEs in General Practice and General Surgery and Medicine with 50 participants. Through analysis, we identified two representations of the patient: the *patient embodied* (where patients are actively involved) and the *patient as*-*a*-*body* (when they are used primarily as a prop for learning). Overall, patients were excluded more during physical examination than talk-based activities. Exclusion occurred through physical positioning of doctor–patient–student, and through doctors and students talking *about*, rather than *to*, patients using medical jargon and online commentaries. Patients’ exclusion was visibly noticeable through eye gaze: patients’ middle-distance gaze coincided with medical terminology or complex wording. Inclusory activities maintained the *patient embodied* during teaching activities through doctors’ skilful embedding of teaching within their care: including vocalising clinical reasoning processes through students, providing patients with a ‘warrant to listen’, allocating turns-at-talk for them and eye-contact. This study uniquely demonstrates the visible nature patient exclusion, providing firm evidence of how this affects patient empowerment and engagement within educational activities for tomorrow’s doctors.

**Electronic supplementary material:**

The online version of this article (doi:10.1007/s10459-016-9688-3) contains supplementary material, which is available to authorized users.

## Introduction

Bedside teaching is a generic phrase that has commonly referred to any type of teaching and learning in the presence of a patient across the full range of modern healthcare settings in which the presence of a bed is not a necessary feature of the encounter (Bleakley and Bligh 2008; Bleakley et al. 2011; Janicik and Fletcher 2003; Steven et al. 2014). This triadic *doctor*–*patient*–*student* interaction within bedside teaching encounters (BTEs) serves the dual purpose of patient care and medical student learning (Celenza and Rogers 2006; Chacko et al. 2007; Wang-Cheng et al. 1989). This dual purpose, however, makes it difficult for educators to marry the two for the benefit of all (Elsey et al. 2014; Hindmarsh 2010): sometimes student learning presides, resulting in patients being objectified, marginalised or side-lined from their own medical consultations (Elsey et al. 2014; Monrouxe et al. 2009; Spencer and McKimm 2010). Such marginalisation runs counter to calls for more active involvement of patients in medical education, where students learn *in* *conjunction* *with* patients rather than merely in their presence (Rees et al. 2007).

### Patient roles within BTEs

Numerous qualitative studies examining patient participation within BTEs, and the roles patients are positioned in or adopt, reveal a spectrum of participation. This spectrum is bookended by the following extremes: from active to passive, included to excluded, full partnership to no involvement, from formally to informally ascribed roles (see Online supplement, S1). However, along with interview studies, the majority of research examining bedside teaching comprises essays and editorials based on personal opinions and experiences (Ramani et al. 2003). Understanding BTEs like this can only offer relatively static insights into patient involvement: they cannot fully capture the full variation of patient participation *as it occurs* during any one encounter. As Spencer et al. argue “[What is] required is more precise descriptions of how exactly patients *are* involved in particular educational settings” (Spencer et al. 2000, p.856). Only a fine-grained analysis of video footage of BTEs can discover *how* patients are interactionally included or excluded during the different phases of BTEs and *how* BTEs are interactionally managed by clinicians in real-time.

### Interactional analyses of BTEs

More recently, using a social constructionist framework viewing power, roles and identities as interactionally constructed, researchers have begun to examine interactional nuances within BTEs (Ajjawi et al. 2015; Elsey et al. 2014; Monrouxe et al. 2009; Rees et al. 2013; Rees and Monrouxe 2008, 2010; Rizan et al. 2014). For example, utilising a dramaturgy analysis of audio data, Monrouxe et al. (2009) examined the extent to which patients were part of the teaching team within hospital based BTEs. They found that patients were interactionally placed into a variety of roles that served to include or exclude them from the team. Patients were excluded by being constructed as a *prop* for students’ learning and as the *audience* to clinicians’ teaching. They were included when constructed as an *actor* through the telling of their illness narratives and as a *director* during physical examinations. No single role could be assigned to any patient across an entire BTE, and no single individual was responsible for the assignment of the roles: rather patients’ involvement in BTEs was constructed and resisted through interaction. Patients were also included/excluded from the teaching team through *frontstage* and *backstage* talk with clinicians using medical jargon in hurried and hushed voices when teaching students, but addressing patients in clearer, louder voices to signal a shift in the encounter from that of teaching to that of patient care. This finding resonates with Hindmarsh’s work considering the dual purpose of service care and student teaching in dental training, in which an interactional separation of service and teaching was found, clearly including and excluding different parties at different stages of the encounters (Hindmarsh 2010; Hindmarsh et al. 2011).

Such strategies, otherwise known as *format changes*, are useful (Elsey et al. 2014). They enable clinicians to manage the interaction during BTEs, address *what is happening now* (or *next*) so that patients and students can follow and contribute appropriately. However, they continue to maintain the divide between student teaching and patient care, rather than moving towards an inclusive and seamless encounter of learning together. Indeed, the main question that now remains is whether these dual purposes can be met simultaneously, or whether patient care and student learning are, indeed, mutually exclusive activity types (Sarangi 2000). The aim of this paper is to examine this issue, drawing on conversation analysis techniques to explore how clinicians manage the dual purpose inherent within BTEs by specifically examining how doctors' and students’ verbal and non-verbal practices work to enhance or limit patient participation within a single ‘activity type’: student feedback (henceforth *feedback*-*in*-*action*).

### Feedback-in-action

The term feedback often refers to comments and judgements evaluating a situation post hoc. However feedback can also be conducted in interaction, and is a crucial aspect of teaching and learning within BTEs that has largely been ignored. These interactional sequences involving feedback-in-action are a fertile environment within which to explore patient involvement: they comprise a seemingly student-centric activity in which performance is continuously evaluated through clinicians’ confirmations and corrections in the presence of the patient. We have recently examined such feedback-in-action within videoed General Practice (GP)-based BTEs (Rizan et al. 2014). Using Mehan’s (1978, 1979) Initiation–Response–Evaluation sequences (I–R–E), we examined how timely and sensitive feedback-in-action from clinicians promotes student learning and development. Indeed, I–R–E components within GP-based BTEs were attributed to participants in the following indicative ways: I = Question/instruction from clinical teacher; R = Reply/action from the student; E = Evaluation of reply/action by clinical teacher. In our corpus feedback sequences were always ‘initiated’ by clinicians who acted as facilitators of the interactions and activities found in the BTEs and predominantly as conduits between students and patients. Evidently, by applying this schema within GP BTEs, no conversational turn was allocated for the patient in this research. However, we found a single instance in which “news” regarding the patient’s future treatment and referral to a specialist was discussed during a feedback sequence, which provided the patient with a *warrant to listen*, that extended beyond the doctor–student dyad (Rizan et al. 2014; Sacks et al. 1978).

Thus, despite the physical presence of patients, they were largely excluded from the analysis. With this in mind, how then do we conceive of patient involvement and participation during these feedback sequences? Given the lack of patient talk, we therefore explore non-verbal aspects of these encounters to examine more precisely the level to which patients are involved within these student-centric activities, thereby addressing limitations of our previous research.

## Method

### Methodological approach

We employ a video ethnographic approach utilising findings from conversation analytic (CA) and ethnomethodological studies of ordinary conversations (Sacks 1995; Schegloff 2007), school classrooms (Macbeth 2003, 2004, 2011; McHoul 1978, 1990; Mehan 1979; Payne and Hustler 1980) and healthcare settings to inform our analysis (Frankel 1990; Heath 1986; Heritage and Maynard 2006; Pomerantz et al. 1995). CA enables us to study participants’ verbal and physical activities. No inference is made regarding participants’ internal state (i.e. cognitively). Rather, we examine the socially constructed, on-going and reflexive (rather than causative) activities within the encounter.

Data was gathered by a single researcher (CE) who was employed specifically to work on the project and therefore had no relationship with the participants outside of the research study. Furthermore, despite any external relationships, we acknowledge that the presence of the researcher and camera will inevitably have an effect on the situation (so-called observer effects), although clinicians were encouraged and actively instructed to adopt their ‘normal’ consultation styles. However, as we do not adopt a positivist perspective in which causality is assumed, where relevant and appropriate the presence and influence of the researcher and camera are accounted for as part of our analysis. For a detailed discussion of observer and camera effects on participant reactions see Heath et al. (2010:37–53).

### Ethical considerations

Ethical approval was granted by two health boards. Age appropriate information sheets and consents forms were developed. The GP and General Surgery and Medicine (GSM) doctors were initially recruited, informed of the study purpose and the research process. In GP settings, when patients telephoned the surgery, reception staff informed them that (a) a medical student might be present for their consultation, (b) the consultation may be recorded as part of a research project; and (c) they could opt into/out of activities at any stage. Within the GSM setting, patients were provided this information on arrival to the clinic. Prior to participation, clinicians, patients and medical students read the information sheet, and signed the consent form if they wished to participate. Opportunities to discuss implications for participating with the researcher (CE) were provided. The present analysis was conducted under the auspices of the original ethics approval.

### Principles of selection and data collection

Previous interactional studies of BTEs are restricted in terms of clinical settings, primarily occurring in single-sites. For this study we therefore involved a range of specialities to include a diverse patient group: GP (n = 12), general surgery and medicine (GSM; n = 15), paediatrics (n = 5) and geriatrics (n = 11). We originally aimed to record equal numbers of BTEs in each setting (with at least two with each clinician), but participation varied due to patient willingness and time factors.

43 BTEs (937 min) were videoed from two angles. The researcher (CE) was present for every BTE session except BTEs 29–32 in which the GP (FD4) elected to operate the cameras herself. We draw on a subset of this data from General Practice (GP: n = 12, 209:20 mn:ss) and General Surgery and Medicine outpatient consultations (GSM: n = 13, 165:20 mn:ss). These settings fit within our broad interpretation of what constitutes bedside teaching. Our rationale for using these subsets is: (1) systematic analysis of feedback sequences was initially conducted on the GP data (12) due to GP-specific funding; (2) the phenomenon of feedback sequences was originally identified in the GSM subset and a systematic analysis of this would be fruitful: in the GP setting there is only ever one student present at a time whereas in GSM there is typical two or more which might have implications for patient involvement; and (3) the GSM and GP subsets have a similar size sample whilst being a distinct medical specialty, suggesting that new findings might be identified.

### Participants

Fifty individuals participated across the 25 BTEs analysed: four GPs, six GSM doctors (8 males, 2 females); two 3rd year students, six 5th year students (3 females, 5 males); 25 patients (8 males) and seven additional parties (e.g. family members, friends: 3 males).

### Analytic approach

Video data was transcribed, anonymised and managed using Transana video management software (Woods and Dempster 2011). Transcriptions included verbal and non-verbal aspects of the interaction, indicated using a modification of Jeffersonian transcription conventions (Jefferson 2004):

- (0.5) = elapsed time in silence (tenths of seconds);
- (.) = a noticeable micro pause (roughly 0.1 s);
- [] = overlapping talk between speakers;
− = a cut off in the talk;
- (word) or () = a possible, but uncertain hearing of the talk;
- (()) = extra descriptions, e.g. laughter, pointing etc.;
- °Hello  = slightly quieter voice.

Further extended conventions for non-verbal features of interactions such as eye-gaze, gestures, positioning, movement and embodiment, developed from prior work (Goodwin 1981, 2003; Heath 1986, 2006), were adapted for our interest in patient involvement (Table 1).

**Table 1:** Transcription conventions for non-verbal activities

Symbol	Explanation
((P → D _____))	Patients’ [P] gaze orientated to [→] doctor [D]. Underscore = gaze length. Double parentheses = beginning/end of gaze
((P → S_____))	Patients’ gaze [P] orientated to [→] student [S]
((P → AP _____))	Patient’ gaze [P] orientated to [→] additional party [AP]
((P↕ _____))	Patients’ gaze [P] oriented to middle-distance [↕] For the purposes of analysis middle-distance meant that the patient was *not* orienting their gaze to either the doctor or medical student
*Description*	Others gestures (e.g. *lowers eyelids, present hands*)

One researcher (AC) reanalysed the GP feedback sequences from Rizan et al. (12) whilst watching and listening to original video data to consider non-verbal activities with respect to patient involvement. Findings were discussed with the team before AC examined all GSM recordings for the presence of feedback sequences. Categorisation was regularly checked and verified with another researcher (CE) for consistency. All three researchers then met, discussed the findings and refined the analysis.

## Results

Ten of 12 GP and eight of the 13 GSM BTEs contained feedback-in-action sequences (n = 108: 100:57 mn:ss). Due to time and funding constraints, 47 I–R–E sequences were then further transcribed and analysed to the level of non-verbal activities (comprising all 8 of the GP excerpts previously published (Rizan et al. 2014) and all 39 from the GSM data). These sequences were embedded within different methods of consultation management by the doctor (Table 2 below), broadly categorised as (a) purely *talk*-*based* activities (i.e. history-taking, diagnostic phases, treatment explanations; n = 23), and (b) *physical examination* activities (also included elements of talk: such as instructions and online commentaries; n = 24). Therefore, the opening phase and related exchanges do not feature in this analysis. However, we have previously examined the opening phase of BTEs in our dataset and document how medical students are introduced to patients (including name and status) and describe how sometimes recaps of patient medical histories are shared if applicable (Elsey et al. 2014).

**Table 2:** Number of examples within talk-based and physicial examination activities across active/passive patient involvement within feedback sequences

Patient involvement within feedback categories	Number
Talk-based	
*Passive patient* Doctor–student I–R–E sequences	10
*Active patient* Doctor–student–patient I–R–E sequences; students talk evaluated and topicalised in doctor–patient talk	13
Physical examination	
*Passive patient* as a prop; as a resource (providing information); as audience (to online commentary for doctor)	20
*Active patient* presenting their body for examination; as recipient of online commentary	4

### Talk-based feedback sequences

This section describes patient involvement during feedback sequences within talk-based phases of consultations: namely history-taking, diagnosis, patient education and treatment planning. Here, patients are excluded through doctors’ and students’ use of medical jargon, talking *about* rather than *to* them and separating teaching from the on-going consultation. Patients are included as key players in students’ education by directly telling them about their illness (rather than this being communicated through the doctor) and through students’ diagnostic/treatment suggestions being topicalised by the doctor when talking with the patient. We now present three indicative in-depth examples of patient exclusion/inclusion within I–R–E feedback sequences.

#### Doctor–student I–R–E feedback sequences (patient as-a-body)

We begin with an example of a GP student–teaching interaction that works to exclude the patient, casting her in a passive role during student learning (BTE 24, Table 3). Here, a 53-year-old female patient presents to the male GP (MD10) with a shoulder problem. This excerpt has previously been discussed in Rizan et al. (2014) but the patients’ presence was rendered ‘invisible’ due to the focus of that analysis being purely talk-based. We now re-present this with the inclusion of non-verbal aspects of the interaction to demonstrate *how* the patient is excluded within this BTE. Interestingly, the feedback sequences take up a large percentage (21:3 mn:ss, 86 %) of this consultation, indicating that student learning takes priority over patient care. To aid the reader’s understanding of the transcripts presented in the rest of this paper the first excerpt (Table 3 below) has been annotated with descriptions of the non-verbal transcript conventions illustrating the different symbols found, with full explanations appearing in Table 1.

**Table 3:**
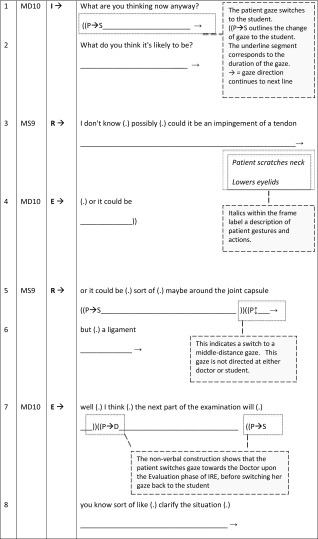
BTE 24, doctor–student I–R–E Sequence (patient as-a-body)

Speaker: MD, Male Doctor; MS, Male (Medical) Student; FP, Female Patient

I–R–E: I, Initiation/Question (of feedback sequence); R, Response/Answer; E, Evaluation

Non-verbal conventions: Follows the verbal timeline and demonstrates the **patient gaze** towards different persons or directions, as well as gestures and movement

In terms of the I–R–E sequence, this excerpt flows as follows: Initiation—MD10 asks question (lines 1–2); Response—Medical student (MS9) provides answer (lines 3 and 5–6); Evaluation—GP evaluates answers (lines 4 and 7–8). Thus, the first evaluation from MD10 (turn 3) provides feedback that MS9’s hesitant response may be correct, implying that an alternative diagnosis is required. MS9 still hesitates in his second response (turn 4), resulting in MD10 providing him implicit correction by suggesting later clarification will occur. Patient exclusion from her consultation is visibalised through the interactional management of the feedback sequence: MS9 physically examines her shoulder, whilst the patient gazes toward him (turn 1 “P → S”), MD10 then asks MS9 to formulate a differential diagnosis (lines 1–2). MS9 pauses from his examination to address MD10’s request (line 3), as he does so, the patient adopts a middle distance gaze (line 3 “P↕”). Her orientation switches to middle-distance again during the students’ second response (line 5) and at the beginning of the evaluation stage (line 7) of the feedback sequence.

Upon closer inspection the patient’s shift in gaze to middle-distance coincides with the production of more specific medical terminology or more complex wording by MS9 (line 3—‘impingement of tendon’ and, line 5 ‘joint capsule but a ligament’). The predominance of technical or medical language indicates that MD10 and MS9 are not orienting their talk towards the patient by modifying their language or explaining the terms (Meehan 1981). The use of medical jargon, along with the use of the *patient as*-*a*-*body* within the IRE feedback sequences, leads to passive patient involvement at this point due to the doctor’s management of this BTE.

#### Doctor–student I–R–E feedback sequences (patient embodied)

Having considered how patients are excluded from student feedback, we now turn our attention to two examples in which the patients are more actively involved in student learning. In the first excerpt the doctor manages the interactional transitions by addressing the patient and the 2 students present during the history-taking, effectively maintaining patient attentiveness throughout. In the second excerpt, we see how feedback sequences can be embedded *within* the history-taking phase of the consultation, thereby achieving a triadic and ‘seamless’ involvement of the patient in student learning.

Table 4 comprises an excerpt taken from the GSM data in which two medical students are present (MS3 and FS3). The patient (a 54-year-old female with musculoskeletal pain: FP8) is incorporated into the BTE by the strategic placing of the feedback sequences by the male doctor (MD5) within the on-going history-taking phase.

**Table 4:**
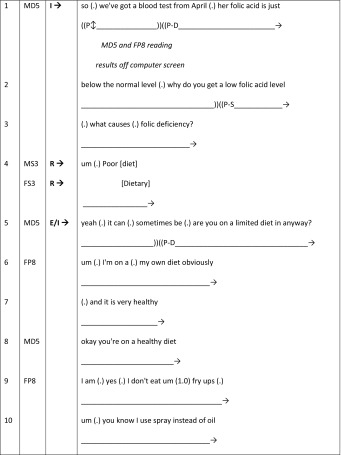

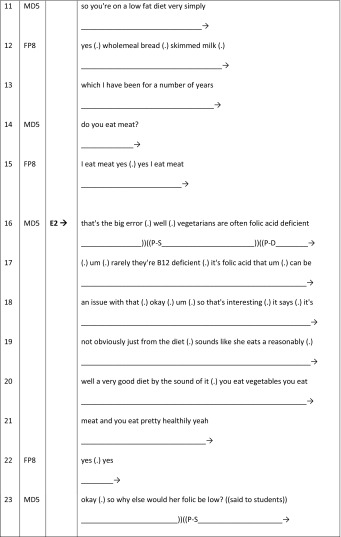
BTE 12, doctor–patient, doctor–student I–R–E sequence (patient active)

This excerpt begins with another classic I–R–E structure, with both medical students simultaneously responding to the initiation question (around “causes of folic deficiency”) produced by MD5 (lines 1–4). MD5 evaluates their response (line 5), confirming this as a possibility and topicalises it by employing it as a basis from which to ask FP8 about her eating habits (lines 5–15). As such the ‘topic’ of the feedback sequences (and therefore students’ responses) are made relevant to the on-going consultation, providing the patient with a ‘warrant to listen’. The BTE continues beyond this excerpt, during which further ‘correct’ student replies then form the next subject for the history-taking between doctor and patient.

Although the evaluation phase (lines 16–22) is primarily intended to teach the students, MD5 constructs his teaching with specific reference to FP8 (note he shifts from “she eats” to “*you* eat”), and uses inclusive language asking the patient to confirm an aspect of the teaching point (lines 20–22). Again MD5 exhibits an awareness of the different parties to his talk. In effect the ‘warrant’ is extended and expanded to create a mediated doctor–patient–student interaction. Throughout this phase, FP8 follows the on-going explanation and clearly shifts her gaze between the students and doctor. The patient does not switch to middle-distance gaze through the evaluation phase. The attentive gaze might be due to the effective management of the consultation, maintaining patient involvement within the BTE via different techniques to aid patient inclusion. However, there is clear interactional separation in terms of participation in that the feedback sequences comprise doctor–student contributions, whereas the history-taking conversation is limited to doctor–patient utterances. This separation is further ‘marked’ by the shift in gaze of MD5 from the students during the feedback sequence, to FP8 throughout the history-taking.

Our final talk-based feedback sequence provides an example of an embedded method. Previous research has demonstrated how patient involvement can be limited through lack of opportunities for patients to have a legitimate voice in the teaching process. Here, we see how the GSM male doctor (MD6) skilfully orchestrates the female patient’s (71-years-old with gallstone problems; FP9) active involvement within student feedback sequences as he facilitates the collaborative telling of the patient’s story during the on-going history-taking phase of the consultation (BTE 14, Table 5). Illustrating how a doctor can elicit patient involvement though both verbal and non-verbal gestures, MD6 uses the patient’s history to build towards a diagnosis *with* the two students (FS2 and FS4) with FP9 being actively involved in the educational process and given a sanctioned evaluative turn-at-talk.

**Table 5:**
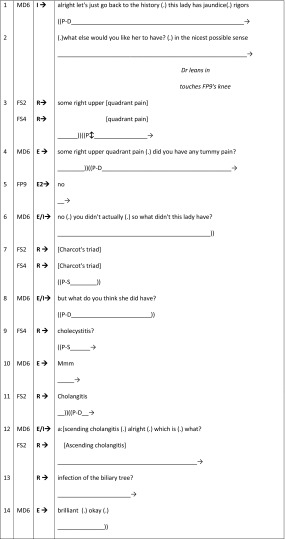
BTE 14, doctor–student–patient I–R–E sequences (patient embodied)

The excerpt begins as MD6 uses humour and non-verbal gestures (eye-contact, leaning in, touching the patient, line 2) to maintain her involvement within this student learning activity. FP9 nods by way of recognition. MD6 continues to include her verbally and non-verbally (lines 4–6), by inviting a contribution during the evaluation phase of the I–R–E sequence. He brings her into the conversation through his positive eye-contact along with his transformation of the students’ technical response to his question (“right upper quadrant pain”, line 3) into lay-language (“did *you* have tummy pain”, line 4). Importantly, focussing on FP9’s gaze, we notice that as the talk adopts more technical language (“upper quadrant pain…” line 3), her gaze shifts away from MD6 to the middle ground (line 3). However, MD6’s purposive eye-contact with FP9 then signals a return in her gaze towards him (line 4). The subsequent transformation of technical to lay language further facilitates her involvement in the form of a secondary evaluation within student-feedback as he acknowledges her primary access to her own experience and the value of this within student learning (line 5). As such the doctor does not over medicalise the opportunistic feedback sequences, and does not reduce FP9 to a teaching resource.

### Physical examination feedback sequences

We now turn to consider how patient involvement and participation occurs during feedback sequences as doctors and students conduct physical examinations on them. Here, we found more exclusionary than inclusionary practices. Thus, patients are often treated as a passive clinical resources and props. Students’ online commentaries about the patients’ condition—reporting positive provisional findings of a physical examination—are produced for the benefit of the doctor rather than the patient. Doctors and students restricting their talk towards patients to merely instructional or information seeking, further inhibits active patient involvement. However, patients are sometimes rendered momentarily active through fleeting verbal ‘side-sequences’: patients being personally addressed to check for comfort or for further information regarding the location of their pain. Additionally, sometimes patients actively present their bodies for examination. Furthermore, students sometimes provide online commentaries for patient, rather than doctor, benefit.

We now present in depth three indicative examples of how patients are excluded and included within I–R–E feedback sequences during physical examinations. We begin with an excerpt in which the patient is afforded no turn in the conversation and is exclusively used as a prop for learning. We then consider two different ways in which online commentaries enable and constrain active involvement of patients in student feedback activities.

#### Doctor–student I–R–E feedback sequences (patient as-a-body)

We begin with an excerpt (Table 6) taken from the GP BTE 24 (Rizan et al. 2014). Here we see the female patient being used by the GP (MD10) as a teaching resource. He renders her passive, in the role of prop, for student (MS9) learning as her elbow joint is the subject of teaching during her consultation. Throughout this sequence the orientation of the various parties is critical to patient involvement: MD10 exclusively talks with MS9, with his body deliberately positioned facing him to facilitate observation of his examination techniques. This doctor–student positioning and the implementation of the I–R–E sequence fails to provide any turns at talk for the patient. The I–R–E sequence is characterised as follows: MD10 turns comprise a series of questions (I) resulting in MS9 responding (R) by either enacting a physical examination or verbally reporting his findings. MD10 evaluates (E) these responses, sometimes through his own verbal/physical actions (e.g. correcting MS9’s positioning of the patient’s arm by physically adjusting it himself without acknowledging her as he does so).

**Table 6:**
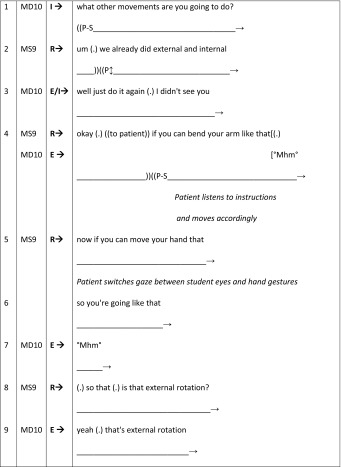

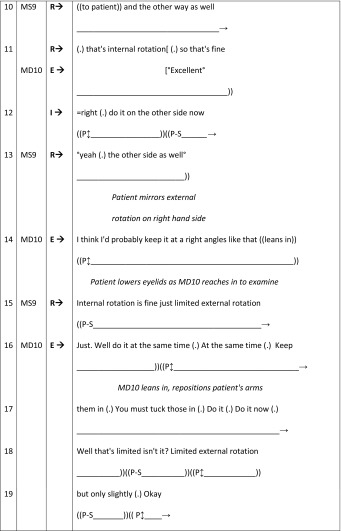
BTE 24, patient as-a-body

Having briefly outlined the doctor–student interaction during this excerpt, we now consider the sequence again, focussing on the patients’ gaze. The scene can be read as two parts. In the first part, MS9 instructs the patient and demonstrates how he wants her to position and move her left arm so that he can check the limits of her external rotation ability. The patient spends much of this time gazing towards him, although she briefly adopts a middle distance gaze when he uses technical language (lines 2–4). The second part of the scene is marked by MD10 asking MS9 to “do it on the other side” (line 12). As he begins to talk, the patients’ gaze adopts a middle ground position, returning to MS9 at the end of his turn. As MS9 softly repeats the instruction (“°yeah (.) the other side as well°”, line 13), the patient takes the initiative to move her right arm. MD10 immediately corrects this action (line 14). However, he is correcting MS9 (for it is his examination) rather than the patient. With this correction he emphasises the patient’s *prop*ness in a number of ways: (1) directing his talk to MS9, despite the patient’s agency in responding; (2) referring to her arm as “it”; and (3) reaching in and physically re-positioning the patient’s arm twice (lines 14 and 16) without first looking at her or asking permission. During this process (lines 14–19), the patient mainly adopts a middle-distance gaze.

Our second example comprises a particular feature of communication within physical examinations: online commentaries. We begin with an excerpt taken from BTE26 (Table 7). The I–R–E sequence begins with the female GP (FD3) asking the male student (MS10) to listen to the patient’s (MP13) chest (I). The response (R) comprises MS10’s examination of MP13 with a minimal evaluation (E) from FD3. Following the initial request from FD3 for MS10 to examine MP13’s chest, MP13 primarily adopts a middle-distance gaze. He briefly looks at MS10 as he consents MP13 for his examination (line 2), but gazes away when he is asked to remove his clothing (line 3). MP13 briefly returns his gaze to MS10 who explains what he is about to do (line 4) and momentarily during the examination (line 7). However, throughout the examination (lines 7–10) MP13 primarily adopts a middle distance gaze as the online commentary produced by MS10 is aimed specifically towards FD3: emphasised by the use of medical jargon (e.g. “42 times 2 (.) it’s 84”, “normal character”) and by talking about, rather than to, the patient (e.g. “his heart rate”, “his lungs”) (Heritage and Stivers 1999).

**Table 7:**
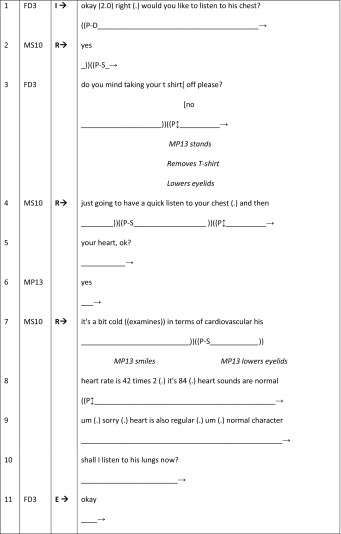
BTE 26, online commentary for the doctor (patient as-a-body)

#### Doctor–student I–R–E feedback sequences (patient embodied)

Continuing the theme of online commentaries, we now present an excerpt from BTE9 (Table 8). This differs significantly from BTE26 in that the commentary is for the benefit of the patient, rather than the doctor (male GSM doctor: MD4). The I–R–E sequence begins prior to this excerpt with MD4 asking the female student (FS2) to examine the female patient’s goitre (I). FS2 responds (R) by performing the examination (line 1) and her findings are confirmed by MD4 (E). In terms of FS2’s actions, as she performs the examination we can see that she satisfies the requirement of MD4 by examining the patient, but she also pays attention to the patient and her concerns through her online commentary. Thus, her commentary begins by addressing MD4 as she reports her finding (using “it” and “she” line 3). However, noticing the patients’ unease (her smile), she quickly shifts to addressing her commentary to the patient (line 4) to inform and reassure her (Heritage and Stivers 1999). Noticeably, there is very little middle-gaze orientation of the patient in this excerpt: she adopts the middle-gaze as she raises her head for the examination, and again when MD4 begins to document their findings (lines 5–6).

**Table 8:**
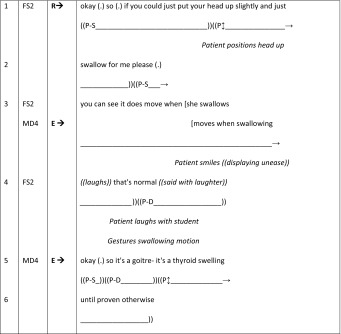
BTE 9, online commentary for patient’s benefit (patient embodied)

## Discussion

The starting point for our paper was to consider student feedback sequences, as a seemingly student-centric activity, within which to investigate patient involvement (Rizan et al. 2014). By their very nature, the default position of feedback sequences is to promote student learning and development in a timely fashion during the course of BTEs: the ‘feedback’ and evaluative utterances of the clinical tutor is principally aimed towards students’ conduct and performance (Pomerantz et al. 1995, 1997). Previous research in this area therefore purely focussed on doctor–student talk and the different strategies used for feedback-in-action within General Practice (GP) settings (Rizan et al. 2014). As patients rarely spoke during these interactions, they were rendered virtually invisible in and by the analysis. We therefore extended this research by documenting how patients are included or excluded from student feedback activities through talk, eye-contact and physical positioning and the consequences of this for patient involvement within the bedside teaching. Thus we utilised the full potential of our video data by exploring patient involvement across two distinct activity types within GP and hospital based BTEs—talk-based feedback sequences and feedback sequences within physical examinations—visually, as well as verbally.

Overall, patients were excluded more commonly during physical examination feedback sequences than during talk-based activities. Exclusion occurred as doctors and students talked about, rather than to patients using medical jargon. The exclusion of patients was visibly noticeable through our observations of patients’ eye gaze: patients shifted their gaze to middle-distance coinciding with the use of more specific medical terminology or complex wording. Although previous research has suggested that such talk might serve to exclude patient involvement within BTEs (Monrouxe et al. 2009), this is the first study to demonstrate the visible nature of this exclusion in terms of how this impacts patient engagement, thereby providing firm evidence of such exclusion.

Patients were also marginalised through the use of online commentaries by students when spoken for the benefit of their clinical educators. Students use these online commentaries to display their understanding and competence to perform different examination techniques and their underlying clinical reasoning. During these commentaries, and doctors’ responses to them, not only do students and doctors use technical terms to describe the patient but they also frequently use impersonal terms, such as referring the patients’ body parts as *it* and talk *about* the patient, “his lungs”, rather than talking *to* the patient. During such talk, patients do not reorient their eye-gaze, maintaining a neutral middle distance throughout. The physical positioning of doctor, student and patient also deters patients’ eye-contact with the doctor and serves to further exclude them from fully participating in the activities.

Previous research examining doctor–patient interaction suggests that eye gaze and an alignment of body posture are an important factor when displaying a mutual engagement during the opening phases of a consultation: this has been called an *engagement framework*, that demonstrates a reciprocated interest in, and attention to, the matters at hand (Goodwin 1981). Indeed, the direction of eye-gaze has been shown to be of utmost importance as a display of attention within doctor–patient interaction (Goodwin 1981; Heath 1986, 2006; Robinson 1998). For example, Robinson (1998) argued that, in primary care settings, doctors are required to simultaneously interact with two different representations of the patient, each providing key information on the patients’ health problems: the *patient embodied* (as an active participant in the consultation), and the *patient inscribed* (in paper documents, or computer records). He examined videoed interactions of primary care physicians and found that doctors conveyed their interest in the patients’ complaint by turning to gaze at the patient, rather than at their medical records. In doing so, the doctor displayed a patient-centered (treating the patient as an expert in their own illness), rather than a doctor-centered (focussing on the doctor’s professional knowledge) orientation.

We argue that within the setting of bedside teaching activities, the doctor’s competing tasks are patient care and student education. These represent the tension between adopting patient-centered and student-centered orientation within the bedside teaching encounter. Drawing on and developing Robinson’s distinctions, we see the two representations of the patient here being the *patient embodied* (during active engagement) and the *patient as*-*a*-*body* (rendered as a prop). Indeed, our data demonstrates clearly how, during physical examinations, patients themselves give up their bodies momentarily for this teaching as they shift to a middle-distance gaze: adopting a psychological distance, they offer the body they *have* rather than the body they *are*. And as the technical talk between student and doctor is not *for them*, patients’ psychological detachment through middle-distance eye-gaze suggests a disengagement activity, during which their interest in, and attention to, matters at hand is not reciprocated (Goodwin 1981). As such, although all three parties are physically present within the BTE, the interaction becomes dyadic (doctor–student), rather than triadic (doctor–patient–student), with patients displaying a compliance or tolerance for student teaching (Chretien et al. 2010).

However, our data has demonstrated that careful management during student teaching can facilitate patient engagement in their education thereby achieving a truly triadic interaction. One notable means of achieving this co-orientation is through establishing a ‘warrant to listen’ for patients and the allocation of turns-at-talk for them within feedback sequences thereby encouraging participation. For example, rather than breaking off from the consultation itself, students were strategically brought into the doctor–patient interaction through the skilful embedding of teaching within patient care. Thus, during the history-taking phase of the consultation, students were included as a way of vocalising the clinical reasoning process. For example, reading aloud the findings from the patients’ blood test, one doctor asked students to hypothesise as to why her folic acid level was low. This provided the patient with a warrant to listen, and so maintained her attention as the students responded. The doctor then topicalised their response in his next enquiry to the patient. Following the patient’s response, during his feedback to the students, he actively included her by addressing her directly: “you eat”. The patient’s turn therefore is significant: it served to evaluate the student’s answers. This demonstrated how patients can be involved in the production of feedback sequences (I–R–E) *if* turns at talk are carefully managed by clinicians. Furthermore, other inclusory activities include a doctor facilitating the collaborative telling of the patient’s story, building towards a diagnosis with the students, through eye-contact, physical positioning and translating technical talk into lay-language. Finally, students’ use of online communication for the benefit of both doctor and patient maintains the active involvement of the patient during teaching episodes, reassuring the patient that her signs are mild (Heritage and Stivers 1999). As a result of these interactional strategies and practices we can reveal how BTEs can move towards triadic (doctor–patient–student) interactions that benefit and involve all parties.

It is essential to pause and consider the contested nature of bedside teaching, its place within modern medical education and its outworking in practice. While some commentators have noted the untimely decline (and even *death*) of bedside teaching (Gonzalo et al. 2010, 2013; Qureshi 2014; Qureshi and Maxwell 2012; Ramani et al. 2003), highlighting the benefits and surmountable impediments (Janicik and Fletcher 2003; Ahmed and El‐Bagir 2002; Nair et al. 1998; Peters and Cate 2013; Qureshi 2014), others have critiqued it as a discourse that is unfit for purpose (Cantillon and Dornan 2014; Glass 1997; Ruffy 1997). While acknowledging these developments in teaching and learning, we remain agnostic about BTEs in terms of their universal suitability or effectiveness for medical student training. It is therefore important to acknowledge that practically marrying patient care and student learning is interactionally complicated and necessarily knotty and requires reflection on the part of the educator. However, it is critical to state the core messages found in this this paper can equally apply in the context of workplace based learning, or indeed any teaching performed in the presence of ‘real’ patients (or otherwise) in any healthcare setting. For example, in this analysis of two subsets from our BTE video corpus only two BTEs fit the description of bedside teaching as contrived, staged or inauthentic interactions (the ‘cold’ medicine Atkinson refers to: Atkinson 1981, 1997). Therefore, the majority of BTEs analysed are actually genuine medical consultations (so-called ‘hot’ medicine) in which actual symptoms, outcomes and treatment decisions are found. Further the challenges identified in this paper remain very real for *any* medical educator in *any* teaching environment (Ajjawi et al. 2015; Bleakley 2006; Bleakley et al. 2011; Dornan et al. 2007; Macbeth 2014).

As with any research, this study has its strengths and challenges. Given our study is grounded in the theory and methods of conversation analysis and ethnomethodology the types of claims that we make are deliberately restricted to the visible/hearable (public) orientations and perspectives that participants exhibit for each other in their talk and actions (Schegloff 1991). To that end we do not feel it is appropriate or consistent to speculate about how common or representative these practices are in medical schools and to make blanket practice recommendations based on a single video corpus. The focus of the current analysis is limited to particular types of interaction within BTEs, namely feedback sequences during teaching exchanges. These types of interaction are inherently more likely to result in a higher concentration of doctor–student utterances and restricted participation opportunities. The analysis presented on physical examinations within BTEs only scratches the surface of the modes of communication that occur in these sensitive and highly embodied activities. Furthermore, although we identified just over 100 min comprising 108 feedback-in-action sequences with 50 participants, we only analysed 47 of these non-verbally due to restricted funding and time. Although this is still a relatively large data set for such an in-depth analysis, the interactions only comprised BTEs with adult patients and doctors across two contexts: GP and GSM. It is possible that if we had analysed a broader spectrum of specialties we might have found further ways in which patients are included and excluded from student feedback processes. For example, had we included data from a paediatric setting in which both patients (the children) and their carers are present, we might have seen more active patient/carer involvement due to the further requirement for students and doctors to achieve full co-operation of the children.

Despite these challenges, our study has strengths. These include the uniqueness of our analysis of both verbal and visual aspects of interaction within a bedside teaching environment: the majority of this research has purely focussed on dyadic doctor–patient interactions, which is not easily transferable to the more complex doctor–patient–student interaction. Additionally, the limited amount of work undertaken within bedside teaching has primarily focussed on talk-in-interaction, largely ignoring the important non-verbal aspects of these encounters (Ajjawi et al. 2015; Elsey et al. 2014; Monrouxe et al. 2009; Rees et al. 2013; Rees and Monrouxe 2008, 2010; Rizan et al. 2014).

Finally, it is important for us to consider what our analysis tells us about medical students’ learning of patient-centeredness. Indeed, during bedside teaching encounters the doctor’s role is to teach students the *what’s* and *how’s* of physicianship including the skills, knowledge, and attitudes required for future practice. Furthermore, while skills and knowledge can be learned outside the clinical environment, patient-centeredness cannot. Consider the dyadic BTE in which patient care and student learning comprise very different and separate activities. Even if doctors role model patient-centeredness during doctor–patient interactions, if this is not translated into practice during student learning activities then students can receive powerful implicit contradictory messages about what patient-centeredness really means, particularly in terms of patient empowerment. These *hidden curriculum* messages prioritise the transmission of the doctors’ professional knowledge to the student: rendering the *patient as*-*a*-*body*, and culminating in a primarily doctor–centred orientation (Goodwin 1981). However, Rees and Monrouxe’s (2010) findings that patients can use humour to resist passive roles in BTEs must considered as a counter-point to this conclusion, even though overall we found little evidence of this practice in our dataset. Therefore it is worth highlighting the humorous on-line commentary exchange in Table 8 (lines 3–4) in which the student responds to the patient’s initial smile resulting in shared laughter, with the doctor not joining in and simply adding the evaluation in next turn.

In a doctor-centred orientation, the students’ purpose within the encounter may be seen as solely procedural, rather than diagnostic: students are primarily there so that they can practice the procedural aspects of physicianship (rather than professionalism), that is the learning and practicing of anatomy, physiology, examination techniques and clinical reasoning in a safe and supported environment. Thus, these actions minimise the focus upon students’ practising appropriate and considerate communication *with the patient*. Doctors’ detached demeanor and actions towards patients during these doctor–student teaching activities also send strong messages to the patients themselves: that they are to yield their bodies without resistance so that tomorrow’s doctors can learn about them. By contrast, the truly triadic doctor–patient–student interaction in which student teaching and feedback is fully embedded within patient care activities prioritise *patient embodiment*, resulting in a seamless role modeling of patient-centeredness, with doctors, students and patients learning together for the benefit of all.

## Electronic supplementary material

Below is the link to the electronic supplementary material.

Supplementary material 1 (DOCX 15 kb)
