# Supplementary material for: Patients embodied and as-a-body within bedside teaching encounters: a video ethnographic study
Source: Adv Health Sci Educ Theory Pract. 2016 May 31;22(1):123–46. doi: 10.1007/s10459-016-9688-3 (PMC5306437; doi:10.1007/s10459-016-9688-3)
Supplement: Supplementary file 1 — Supplementary material 1 (DOCX 15 kb) [file 10459_2016_9688_MOESM1_ESM.docx]

**Appendix - Supplementary material**

Online Table 1 (S1): Patient involvement – available roles for patients within BTEs

| Type of role | Definition | Literature sources |
| --- | --- | --- |
| Passive | This term covers a variety of patient roles within BTEs including the patient as:  Data, example, case (e.g. signs, symptoms, conditions)  Teaching material or aid  Teaching resource  Clinical object or body, especially for students to practise on during physical examinations (e.g. prop)  As a result the involvement of the patient within is often ‘opportunistic’ ‘one-off’ events, on the part of the clinician, with teaching/learning the primary purpose of the interaction. | Cox (ii) 1993; Linsfor and Neelon 1980; ;; M^c^Lachan et al. 2012; Monrouxe et al 2009; Ramani 2008; Rees et al. 2007; Spencer et al. 2000; Spencer and McKimm 2010; Stark et al. 2008; Towle et al 2010; Wykurz and Kelly 2002 |
| Tolerant | Patient merely accepts physical examination as par for course in teaching hospital | Chretien et al 2010 |
| Excluded from interaction | Use of technical or medical language between the clinician and medical student means that patient does not fully understand what is being said, nor is it explained to them in clear, lay language | Bellet 1997; Fletcher et al 2005; 2007; Lehmann et al 1997; Janicik and Fletcher 2003; Linsfor and Neelon 1980; Ramani et al. 2003; Spencer and McKimm 2010 |
| Social | Patients benefit from human contact when in hospital, to break up the boredom and feelings of isolation | Chretien et al 2010; Rees et al. 2007 |
| Helping | Patients are willing to give something back to the health service that has treated them and derive value from contributing to future doctors | Chretien et al. 2010; Rees et al. 2007; Spencer et al. 2000 |
| Learning | During BTEs patients are educated and informed about their illnesses, treatments, reasons for being in hospital and what happens next etc. Promotes patient understanding. | Chretien et al 2010; Fletcher et al. 2005; Lehmann et al. 1997; Spencer et al. 2000 |
| Whole patient | Moving beyond treat patients as bearers of  Patient as an individual. Understanding the patient within the social setting, local context and social structures | Boehm 2008; Branch et al. 1991; Fletcher et al. 2007; Spencer et al. 2000; Weissmann et al. 2006; |
| Patient teachers, experts and educators | Patient s have a ‘unique illness experience’ (Stacy and Spender 1999:693), which makes them ‘experts’ on their conditions and health issues and ideal instructors in community settings.  ‘Patient is given preparation for specific teaching role, may actively question students, may be involved in giving feedback and evaluating students’ performance’ (Towle et al. 2010: 66). This role suits the development of students’ physical examination techniques and communication skills. | Stacy and Spencer 1999; Towle et al. 2010; Wykurz 1999; |
| Partners | Patients are fully involved in BTEs, planning teaching sessions, student assessment, curriculum development/enactment, employed by medical schools | Spencer et al. 2011; Spencer and McKimm 2010; Towle et al. 2010 |

**Online table (S1) additional references**

Bellet, P.S. (1997). Bedside presentations and patients' perceptions of their medical care. *New England Journal of Medicine,* 337, 714-715; author reply 715-716.

Boehm, F.H. (2008). Teaching bedside manners to medical students. *Academic Medicine,* 83, 534.

Branch, W.T., Arky, R.A., Woo, B., Stoeckle, J.D., Levy, D.B., & Taylor, W.C. (1991). Teaching medicine as a human experience: a patient-doctor relationship course for faculty and first-year medical students. *Ann Intern Med,* 114, 482-489.

Chretien, K.C., Goldman, E.F., Craven, K.E., & Faselis, C.J. (2010). A Qualitative Study of the Meaning of Physical Examination Teaching for Patients. *Journal of General Internal Medicine,* 25, 786-791.

Cox, K. (1993). PLANNING BEDSIDE TEACHING .2. PREPARATION BEFORE ENTERING THE WARDS. *Medical Journal of Australia,* 158, 355-357.

Fletcher, K.E., Furney, S.L., & Stern, D.T. (2007). Patients speak: what's really important about bedside interactions with physician teams. *Teaching & Learning in Medicine,* 19, 120-127.

Fletcher, K.E., Rankey, D.S., & Stern, D.T. (2005). Bedside interactions from the other side of the bedrail. *Journal of General Internal Medicine,* 20, 58-61.

Janicik, R.W., & Fletcher, K.E. (2003). Teaching at the bedside: a new model. *Medical Teacher,* 25, 127-130.

Lehmann, L.S., Brancati, F.L., Chen, M.C., Roter, D., & Dobs, A.S. (1997a). The effect of bedside case presentations on patients' perceptions of their medical care. *New England Journal of Medicine,* 336, 1150-1155.

Lehmann, L.S., Brancati, F.L., & Dobs, A.S. (1997b). Bedside Presentations and Patients' Perceptions of Their Medical Care. *New England Journal of Medicine,* 337, 714-716.

Linfors, E.W., & Neelon, F.A. (1980). Sounding Boards. The case of bedside rounds. *New England Journal of Medicine,* 303, 1230-1233.

McLachlan, E., King, N., Wenger, E., & Dornan, T. (2012). Phenomenological analysis of patient experiences of medical student teaching encounters. *Medical Education,* 46, 963-973.

Monrouxe, L.V., Rees, C.E., & Bradley, P. (2009). The Construction of Patients' Involvement in Hospital Bedside Teaching Encounters. *Qualitative Health Research,* 19, 918-930.

Ramani, S. (2008). Twelve tips for excellent physical examination teaching. *Medical Teacher,* 30, 851-856.

Ramani, S., Orlander, J.D., Strunin, L., & Barber, T.W. (2003). Whither bedside teaching? A focus-group study of clinical teachers. *Academic Medicine,* 78, 384-390.

Rees, C., Knight, L., & Wilkinson, C. (2007). “User Involvement Is a Sine Qua Non, Almost, in Medical Education”: Learning with Rather than Just About Health and Social Care Service Users. *Advances in Health Sciences Education,* 12, 359-390.

Spencer, J., Blackmore, D., Heard, S., McCrorie, P., McHaffie, D., Scherpbier, A., et al. (2000). Patient-oriented learning: a review of the role of the patient in the education of medical students. *Medical Education,* 34, 851-857.

Spencer, J., Godolphin, W., Karpenko, N., & Towle, A. (2011). Can patients be teachers? Involving patients and service users in healthcare professional's education. London: The Health Foundation.

Spencer, J., & McKimm, J. (2010). Patient involvement in medical education. In T. Swanwick (Ed.), *Understanding medical education: evidence, theory and practice* pp. 181-194). Chichester: Wiley-Blackwell.

Stacy, R., & Spencer, J. (1999). Patients as teachers: a qualitative study of patients' views on their role in a community-based undergraduate project. *Medical Education,* 33, 688-694.

Stark, P., Bax, N., Spencer, J., & Hague, M. (2008). Involving patients as educators in undergraduate education. (p. 6). University of Sheffield.

Towle, A., Bainbridge, L., Godolphin, W., Katz, A., Kline, C., Lown, B., et al. (2010). Active patient involvement in the education of health professionals. *Medical Education,* 44, 64-74.

Wang-Cheng, R.M., Barnas, G.P., Sigmann, P., Riendl, P.A., & Young, M.J. (1989). Bedside case presentations: why patients like them but learners don't. *Journal of General Internal Medicine,* 4, 284-287.

Weissmann, P.F., Branch, W.T., Gracey, C.F., Haidet, P., & Frankel, R.M. (2006). Role Modeling Humanistic Behavior: Learning Bedside Manner from the Experts. *Academic Medicine,* 81, 661-667.

Wykurz, G. (1999). Patients in medical education: from passive participants to active partners. *Medical Education,* 33, 634-636.

Wykurz, G., & Kelly, D. (2002). Developing the role of patients as teachers: literature review. *BMJ,* 325, 818-821.
